# Therapeutic Modulation of the Host Defense by Hemoadsorption with CytoSorb^®^—Basics, Indications and Perspectives—A Scoping Review

**DOI:** 10.3390/ijms222312786

**Published:** 2021-11-26

**Authors:** Thomas Köhler, Elke Schwier, Janina Praxenthaler, Carmen Kirchner, Dietrich Henzler, Claas Eickmeyer

**Affiliations:** 1 Department of Anesthesiology, Surgical Intensive Care, Emergency and Pain Medicine, Ruhr University Bochum, Klinikum Herford, 32120 Herford, Germany; elke.schwier@ruhr-uni-bochum.de (E.S.); janina.praxenthaler@ruhr-uni-bochum.de (J.P.); dietrich.henzler@ruhr-uni-bochum.de (D.H.); claas.eickmeyer@ruhr-uni-bochum.de (C.E.); 2 Department of General and Visceral Surgery, Thoracic Surgery and Proctology, Ruhr University Bochum, Klinikum Herford, 32120 Herford, Germany; carmen.kirchner@ruhr-uni-bochum.de

**Keywords:** hemoadsorption, CytoSorb^®^, amount of blood purified, COVID-19, immune system, cytokines, cytokine storm, sepsis, septic shock, hemophagocytic syndrome

## Abstract

The “normal” immune response to an insult triggers a highly regulated response determined by the interaction of various immunocompetent cells with pro- and anti-inflammatory cytokines. Under pathologic conditions, the massive elevation of cytokine levels (“cytokine storm”) could not be controlled until the recent development of hemoadsorption devices that are able to extract a variety of different DAMPs, PAMPs, and metabolic products from the blood. CytoSorb^®^ has been approved for adjunctive sepsis therapy since 2011. This review aims to summarize theoretical knowledge, in vitro results, and clinical findings to provide the clinician with pragmatic guidance for daily practice. English-language and peer-reviewed literature identified by a selective literature search in PubMed and published between January 2016 and May 2021 was included. Hemoadsorption can be used successfully as adjunct to a complex therapeutic regimen for various conditions. To the contrary, this nonspecific intervention may potentially worsen patient outcomes in complex immunological processes. CytoSorb^®^ therapy appears to be safe and useful in various diseases (e.g., rhabdomyolysis, liver failure, or intoxications) as well as in septic shock or cytokine release syndrome, although a conclusive assessment of treatment benefit is not possible and no survival benefit has yet been demonstrated in randomized controlled trials.

## 1. Introduction to Hemoadsorption

Sepsis and septic shock are complex, life-threatening conditions with persistantly high [1] multiorgan failure-related mortality of up to 70%. Sepsis is the inglorious most common cause of death in critical care and one of the greatest challenges for healthcare systems worldwide [2,3,4,5].

Multiorgan failure in sepsis is predominately caused by dysfunctional microcirculation [6], induced and regulated by multiple humoral and cellular mechanisms. The different components of the immune system that are organized in highly complex, dynamic network-like structures are not well-understood [7]. The fact that sepsis and other inflammatory conditions are not uniform, but inter-individually distinct, causes clinically variable phenotypes of inflammatory states with alternating pro- and anti-inflammatory characteristics [8].

Conventional treatment includes early anti-infective use, volume resuscitation, and catecholamine therapy for hemodynamic stabilization and extracorporeal organ support, such as renal replacement therapy (RRT) [9]. Ideally, the dysregulated immune response is controlled and “immunologic homeostasis” is restored [10]. CytoSorb^®^ is the most widely used hemoadsorption procedure at present [11] that specifically targets hyperinflammation by extracorporeal removal of pro-inflammatory substances, i.e., cytokines.

## 2. Methods

This scoping review was conducted in compliance with PRISMA-ScR [12]. Although less rigorous in methodology than a systematic review, the scoping review enjoys the benefit of answering important questions in the absence of highly graded evidence to the significance of hemoadsorption with CytoSorb^®^. We aimed to provide the clinician with an orientation to the state of research, to outline corresponding topic areas, and, without judging the methodological quality, to map the often-incomplete evidence. Our aim was to simplify the decision-making-process in practice for the intensive care physician at the bedside.

A selective literature search was performed in the PubMed database from January 2016 until 31 May 2021. The items used were: *Basic of Immune System, CytoSorb, Hemoadsorption, Cytokine, Cytokine Storm, Sepsis, Septic Shock, and Hemophagocytic Syndrome*. In addition, the database https://literature.cytosorb-therapy.com/ (accessed on 31 May 2021), which is freely available on the Internet, was included in the literature search to identify publications in sources not listed in PubMed.

English-language and peer-reviewed publications from January 2016 to May 2021 were included. In a few exceptions, seminal literature from before 2016 was also included. This was done after internal review if an important effect on overall understanding was expected.

**Figure 1:**
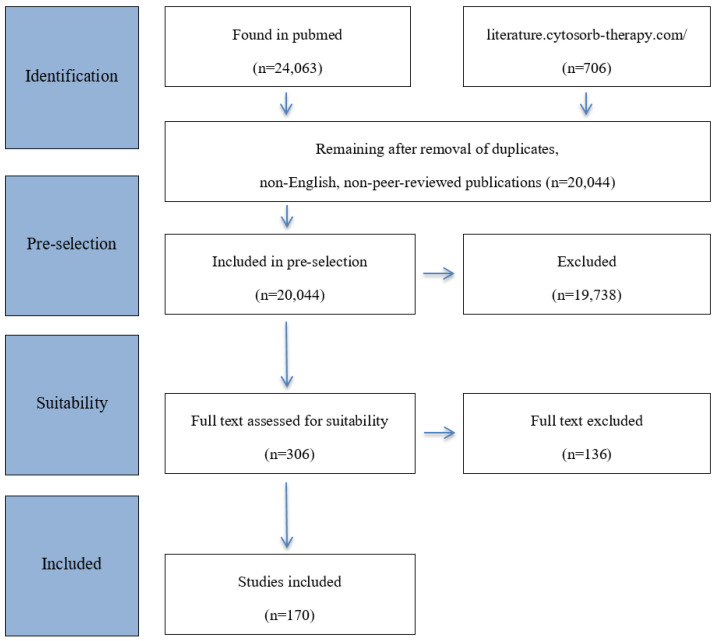
Flowchart for study selection adapted from the PRISMA-ScR statement [12].

The primary publications found in the database search were then subjected to an internal multistep selection process. First, duplicates were removed. Selection was based on heading, keywords, and abstract, and weighted by publication date and context. Then, two by two experienced reviewers classified and included relevant publications (Figure 1) based on the following key criteria:

- Infection/Host response
- Regulation and dysregulation of the immune system
- Cytokines and cytokine storm
- Corona-Virus induced Disease 2019 (COVID-19)
- Hemoadsorption with CytoSorb^®^—basic principles and function
- Hemoadsorption—Indications
  ○ Systemic Inflammatory Response Syndrome (SIRS), sepsis and septic shock
  ○ Trauma-induced inflammation (trauma, rhabdomyolysis)
  ○ Liver failure, hyperbilirubinemia
  ○ Acute respiratory distress syndrome (ARDS)
  ○ Extracorporeal membrane oxygenation (ECMO)
  ○ COVID-19-associated ARDS (CARDS)
  ○ SIRS, perioperative use, cardiac surgery
  ○ Intoxications
  ○ Side effects
  ○ Dosage of antibiotics

For these publications, the full text then assessed for its suitability with respect to the objective of the review and information was included accordingly. Each publication was evaluated according to the following criteria: (patho-)physiological basics, case reports, case series, letter, retrospective studies, prospective studies, and reviews. In order to provide an overview of the current state of clinical use, we deliberately refrained from a strict assessment of study quality and partially included publications of lower evidence (e.g., case reports).

The respective article assigned to the first appropriate group, so that, for example, the frequent formulation “Hemoadsorption with CytoSorb^®^” required a grouping in the chapter “Hemoadsorption”. 

Finally, after extensive discussion among all authors, the texts assessed with respect to various factors (e.g., publication date, methodology, results, and impact) and summarized separately below for each topic.

## 3. Infection Response and Immune System Regulation

Injury or infection initially leads to local activation of humoral factors (e.g., complement factors) and activation of the innate immune system, e.g., via pattern recognition receptors (PRR’s). These are diverse sets of receptors located on the surfaces or internally of various cells of the immune system. They recognize both pathogen- and damage-associated molecular patterns (PAMPs/DAMPs). PAMPs (e.g., Staphylococcus aureus Toxic Shock syndrome Toxin and Clostridium perfringens toxin) are products formed by various microbes. DAMPs, released by damaged endogenous cells or formed during the processing of extracellular matrix, can trigger inflammatory reactions as alarmins via the induction of cytosolic multiprotein complexes, so-called inflammasomes with subsequent, e.g., gasdermin-D induced, proinflammatory cell death called pyroptosis [13,14,15,16].

### 3.1. Cytokines and the Cytokine Storm

Cytokines control cell proliferation and differentiation, the regulation of angiogenesis, and immune and inflammatory responses. Characteristic plasma cytokine profiles show TNFα, IL1, IL8, and MCP-1 peaks in the first minutes to hours after infection, followed by an IL6 increase [17,18]. IL10 is released later to control the inflammatory response [16,19].

Cytokines are key components to inflammation and their temporal and spatially fine-tuned interplay with, i.e., the cellular components of the immune system will most likely result in controlled inflammation with subsequent healing. However, severe injury and disease (Table 1) can cause overproduction of several cytokines that may cumulate in systemic inflammatory response syndrome (SIRS) and which is characterized by four criteria: tachypnea, tachycardia, leukopenia or leukocytosis (leucocyte count > 12,000 cells/μL or <4000/μL), and fever or hypothermia (body temperature > 38 °C or <36 °C). Although this syndrome presents itself inter-individually in very different degrees the SIRS criteria still seem useful to indicate a massive activation of the host defense triggered by, e.g., an infection [20].

The term “cytokine storm” describes a very heterogeneous group of diseases characterized by excessive, life-threatening, potentially fatal hyperinflammation [21]. Once triggered, the massive release of proinflammatory cytokines as part of the early immune response causes shock and organ failure by microcirculatory disturbances and coagulopathy [9]. (Para-) clinical findings include: persistent fever, cytopenia, splenomegaly, hepatitis, or coagulopathies [22,23].

Infectious and non-infectious diseases such as active rheumatic diseases can cause a cytokine storm syndrome with massively elevated IL6 levels. [22]. In contrast, hemophagocytosis syndromes are characterized by high IFNγ and IL10 levels in combination with mildly elevated IL6 [24], an “anti-inflammatory cytokine storm with early immunosuppression”.

The pathophysiological response to the cytokine storm varies interindividually [17]. Elevated IL6 and resistin levels correlate with sepsis severity as well as end-organ damage and appear to be associated with increased mortality [25]. In general, high concentrations of proinflammatory cytokines are associated with increased mortality [26,27,28]. Elevated anti-inflammatory cytokines, such as IL10, are also predictive of sepsis severity and worsened outcome [28,29]. Relevant Cytokines are listed in Table 2.

A severe, potentially fatal cytokine storm may possibly occur in association with COVID-19, leading to the excessive release of various pro- and anti-inflammatory cytokines (e.g., IL1, IL2, IL6 IL7, IL10, GCSF, IP10, MCP1, MIP1A, INFy, and TNFα) [33,34,35,36,37,38,39].

However, a recent, critically discussed rapid review [40] concluded that IL6, an important proinflammatory cytokine, synthesized in fibroblasts, monocytes, T-cells, and endothelial cells [41], is lower in patients with severe COVID-19 compared with other hyperinflammatory states associated with acute respiratory distress syndrome (ARDS), sepsis, or cytokine release syndrome (CRS) [42]. On the other hand, two randomized controlled trials (ReMAPCap and RECOVERY) showed that blockade of the IL6 pathway with tocilizumab improves the prognosis of COVID-19 patients [43,44]. In conclusion, the data remain controversial and it appears that the often-described hyperinflammation linked to COVID-19 is by no means the only pathomechanism [42].

### 3.2. Immunosuppression

To control inflammation, anti-inflammatory cytokines are increasingly released as the immune response progresses. Antigen presenting cell (APC) activation levels, expression of HLA-DR, and co-stimulatory molecules decrease. Antigen presentation, pro-inflammatory mediators, and phagocytosis are reduced. In consequence, an anti-inflammatory situation (IL10↑↑, APC↓) known as Sepsis Associated Immunosuppression (SAI) or Injury Associated Immunosuppression (IAI) may develop (Table 1). [14,45,46,47,48].

Major players in SAI may be myeloid-derived suppressor cells (MDSC). They mediate T-cell dysfunction, resulting in a higher incidence of secondary infections [48,49,50,51]. Another cellular entity contributing to SAI are regulatory T-cells (Tr1 cells), that, among other things, inhibit the activation of key inflammasomes (Nod-like receptor protein: pyrin domain containing 3 (NLRP3) inflammasome) through increased IL10 release [16,52]. Human leucocyte antigen (HLA-DR) plays an important role in T-cell activation as a key MHC class II molecule on monocytes/macrophages. HLA-DR establishes together with various co-factors (e.g., CD40-CD40L) the link between antigen-presenting cells and the T-cell receptor (TCR). The expression of HLA-DR is downregulated by the release of IL10. This may contribute to immunosuppression when IL10 levels are excessively high. As a clinical example, frequent immunosuppression with increase of infection (e.g., pneumonia) after CNS injury is (co-)caused by a reduced HLA-DR level (cut off: <8000 molecules/cell) longer than two days [14].

Based on a better understanding of the interplay of pro- and anti-inflammatory cytokines by overlapping, in part redundant, networks of cells and cytokines, new therapeutic approaches have been developed that, as with hemoadsorption, aim to modulate the amount of inflammatory cytokines as part of the host defense [34,35,38,53].

## 4. Hemoadsorption with CytoSorb^®^

### 4.1. Basics

To control a dysregulated immune response has long been the subject of various therapeutic efforts. The rational is to reduce the elevated concentrations of pro-and anti-inflammatory cytokines equally, with preserving their ratios, instead of intervening at a specific pathway in the complex immunological network [7,30]. Hemoadsorption is based on this therapeutic principle and aims to restore the immunologic balance [10] by reducing the plasma concentrations of *pro*- and *anti*-inflammatory mediators below a “toxic threshold” [54]. It is also possible that the change in the equilibrium of cytokines between the affected tissue and the blood plays an important role. As the concentration gradient increases, chemotaxis-mediated increased migration of immunocompetent cells into the affected region appears possible [55].

CytoSorb^®^ (CytoSorbents Corporation, Monmouth Junction, NJ, USA), approved in Europe in 2011 for patients with excessive cytokine levels, is a high-tech polymer adsorbent with a total surface area of over 45,000 sqm and very high binding capacity that is applied for blood purification in an extracorporeal circuit. Highly porous polyvinylpyrrolidone-coated polystyrene-divinylbenzene beads (polymer beads with a size of: 300–800 µm) [41] bind through a combination of hydrophobic interactions, van der Waals’ forces, and charge-induced interactions a broad spectrum of molecules with a molecular weight <55 kDa. Various hydrophobic, pro-and anti-inflammatory mediators, immune response-triggering DAMPs or PAMPs, as well as endogenous metabolism-generated (e.g., bilirubin and myoglobin) or pharmacologic substances (e.g., anticoagulants and psychotropic drugs) are adsorbed [56,57,58,59,60,61,62]. Concentration is a major determinant of adsorption efficiency, meaning higher concentrations lead to faster adsorption [61,63].

The CytoSorb^®^ adsorber is predominantly and in combination used with continuous renal replacement therapy. Operation as an additional hemoperfusion with any approved blood pump system is possible as well as integration into an extracorporeal membrane oxygenation (ECMO) circuit [64,65,66]. The required anticoagulation can be managed systemically with heparin or locoregionally with citrate [41,67,68].

### 4.2. Indications

Hemoadsorption with CytoSorb^®^ may be used for the treatment in numerous indications (Table 3).

Hemoadsorption with CytoSorb^®^ requires an extracorporeal circuit, e.g., in the context of CRRT, ECMO or as a standalone procedure in the context of hemoperfusion. All these procedures have specific complications. In addition, the complications due to the intravascular access devices themselves must be considered.

If the CytoSorb^®^ Adsorber is used as an adjunctive procedure, e.g., in septic shock or as part of ECMO treatment, these factors do not play a role. However, if primary CytoSorb^®^ therapy is planned, the intensive care therapist must carefully consider the complications associated with the procedure when determining the indication. From the authors’ point of view, the use of CytoSorb^®^ in the context of acute, time-critical treatment of intoxications (Table 4), life-threatening bleeding under DOAC [59], or acute rhabdomyolysis [60], e.g., in the context of malignant hyperthermia, is sufficiently well documented and should not be withheld from the patient, i.e., in these cases the establishment of an extracorporeal circuit, usually a CRRT, seems justified.

In other situations (Table 3), we are very critical of the establishment of an extracorporeal circuit to perform hemoadsorption and consider the current data situation in this regard to be insufficient.

#### 4.2.1. SIRS, Sepsis and Septic Shock

The adjunctive therapy of SIRS, sepsis, or septic shock with CytoSorb^®^ should help to reduce, downregulate, or prevent an excessive immune response (“cytokine storm”). Capillary leakage and vasoplegia should be alleviated and the microcirculation improved [70]. Since no direct measurements of efficacy are available, a decrease in IL6-concentration and vasopressor requirement, or an increase in lactate clearance have been suggested as surrogate parameters [71]. A decrease in lactate of >2 mmol/L and vasopressor requirements, i.e., to below 20% of the initial dose [70], are supposed to indicate therapeutic success [65,71] whereby the duration of treatment should depend on the individual patient’s response [41]. Several in vitro studies exhibited effective adsorption of a broad spectrum of PAMPs and DAMPs or various cytokines by CytoSorb^®^. Procalcitonin is equally adsorbed, which must be taken into account in the interpretation of lab test results [30,32]. However, the IL6 concentration, which is frequently used to indicate start and efficacy of hemoadsorption therapy and for the assessment of the effect of hemoadsorption, has recently been questioned for its usefulness since the levels are balanced by the endogenous turn-over and the extracorporeal elimination [72,73,74]. CytoSorb^®^ therapy could be useful in septic shock [75,76], but indication, indication thresholds and duration of therapy are controversially discussed. With explicit attention to the limitations of data from animal studies, especially the conditional applicability to humans, Peng et al. demonstrated prolonged survival in the rat model under hemoadsorption [77]. A randomized controlled trial demonstrated no mortality reduction for hemoadsorption in ARDS patients with low case severity (APACHE II: 23) [78]. Methodologically, the study was criticized for its short, discontinuous CytoSorb^®^ use (6 h/d for 7 d) which did not significantly reduce IL6 levels. In contrast, data from the CytoSorb^®^ registry reported a mean CytoSorb^®^ application duration of 50 h [79]. Brouwer et al. reported a significant reduction of mortality in patients with septic shock if continuous CytoSorb^®^ therapy was applied for 56 h [65]. However, the statistical method of “stabilized inverse probability of treatment weigths (sIPTW)” has been scrutinized [80]. Probably, the application of a higher “dose” (amount of blood purified (ABP), unit: l blood/kg bw), higher blood flow, in combination with a longer treatment (ca. 85 h), is associated with lower mortality [72]. More retrospective studies demonstrated a mortality benefit for hemoadsorption [81], especially if CytoSorb^®^ therapy was started early (within 12 h) [82]. On the other hand, several recently published studies have failed to demonstrate any effect of hemoadsorption on mortality [74,83].

The optimization of application duration, adsorber exchange modalities, and blood flow rate may be crucial for success and is subject of intensive research.

However, clear evidence of a survival benefit for CytoSorb^®^ therapy in the form of a randomized, prospective study (RCT) is still missing.

#### 4.2.2. Trauma Induced Inflammation, Injury Associated Immunosuppression and Rhabdomyolysis

Various injuries ((poly-) trauma, burns, and major surgery) can lead to massive SIRS with, e.g., increased DAMPs-induced expression of pro- and anti-inflammatory mediators, occurring especially within the first 24 h [84,85]. Individually different, a phase of immune paralysis, the IAI follows. CytoSorb^®^ may offer a therapeutic approach here, via control of the massive release of various DAMPs (e.g., high mobility group box-1 protein (HMGB1) or extracellular histones) [86]. Trauma is often aggravated by muscle injury and reperfusion syndrome with subsequent rhabdomyolysis. This may also be caused by reperfusion syndrome following occlusion of large vessels. Non-traumatic diseases (malignant hyperthermia, autoimmune diseases, and intoxications) may also be causative [87]. Myoglobin precipitation (haemoprotein MG17.8 kDa) in the renal tubules (crush kidney), combined with elevated concentrations of free oxygen radicals [88], can damage the kidneys to the point of acute failure (ARF) [89]. Important therapeutic measures include early aggressive volume therapy, consistent surgical therapy for compartmental syndromes [90], and, if necessary, RRT [91]. However, RRT cannot prevent ARF [90]. CytoSorb^®^ rapidly lowers myoglobin serum levels due to its high myoglobin extraction rate and thus can be recommended as part of a multimodal treatment approach [87,88,92]. It remains unclear whether CytoSorb^®^ can prevent ARF in rhabdomyolysis.

#### 4.2.3. Liver Failure and Hyperbilirubinemia

In up to 40% of critically ill patients, hyperbilirubinemia is present with an increased risk of death [93]. Clinical and paraclinical findings typically include varying degrees of icterus, lactic acidosis, coagulation disorders, hepatic encephalopathy, and circulatory insufficiency. ARF is an additional complication. Acute or acute-on-chronic liver failure (ALF/ACLF) can be primary (e.g., viral) or secondary (e.g., cholestasis-, hypoxemia-, or shock-related). It is based on a complex immunopathology involving the release of various PAMPs and DAMPs, as well as the release of a variety of cytokines and the activation of various immune cells such as monocytes and macrophages. Frequently, localized hepatic inflammation leads to generalized vasopathy and multiorgan failure via SIRS [94,95].

In addition to water-soluble ammonia, hydrophilic direct bilirubin and bile acids, the accumulation of indirect, albumin-bound, i.e., hydrophobic bilirubin plays an important role in the pathogenesis of acute liver failure [57,96]. Progression to ALF or ACLF may be perpetuated by “hepatic SIRS” [97]. CytoSorb^®^ adsorbs indirect bilirubin and bile acids and modulates the concentrations of involved cytokines [57,98] while RRT controls serum concentrations of ammonia and direct bilirubin [99,100]. With CytoSorb^®^, bilirubin is released from its strong albumin binding and gets adsorbed while albumin concentrations remain virtually unchanged [56,57], resulting in an overall improvement in liver function [41]. This is true even when used for several weeks [62]. CytoSorb^®^ therapy may be a simple, user-friendly alternative to bridge to functional recovery or orthotopic liver transplantation [93,101]. Initial clinical trial results appear positive [69].

#### 4.2.4. Acute Respiratory Distress Syndrome (ARDS) and Extracorporeal Membrane Oxygenation (ECMO)

Hallmarks of ARDS, one of the most common diagnoses in intensive care units [102], are acute inflammation with increased pulmonary vascular permeability, increased lung weight, and loss of aerated lung tissue [103]. The main symptom is rapidly progressive respiratory distress coupled with a “cytokine storm” that develops with the typical course of pro- and anti-inflammatory cytokine peaks [17,104].

Specific therapeutic procedures do not yet exist. Rather, evidence-based treatment is characterized by supportive therapeutic procedures. These include, for example, lung-protective ventilation [105], adequately high PEEP levels [106], prone positioning [107,108], or the establishment of extracorporeal membrane oxygenation (ECMO) [109,110].

In addition to the underlying disease, the foreign surface of the ECMO circuit itself, rapidly amplifies hyperinflammation by complement, endothelial, and leukocyte activation of varying degrees [66,111,112], making CytoSorb^®^ therapy a rational option. Several studies have reported a decrease in extravascular lung water (normalization of pulmonary vascular permeability), vasopressor requirements [104,113], and inflammatory parameters, associated with rapid clinical stabilization [114]. The high ECMO blood flow rates have a dose-increasing, effect-enhancing, and thus, if high enough (ABP > 13 L/kg), possibly mortality-lowering effect [72].

#### 4.2.5. COVID-19-Associated ARDS (CARDS)

In COVID-19, a potentially massive cytokine storm may cause lethal destruction of the lung with attenuated pulmonary vasoregulation, ventilation-perfusion mismatch, and high risk of thrombosis and impairment of other organ functions via microvascular damage (endothelitis) as well [33,35,36,37,53,115]. This may explain the phenotypic differences between “typical” ARDS and CARDS characterized by hypoxemia, normal compliance, and altered ventilation-perfusion ratio [116,117]. Based on pathophysiological considerations, some authors recommend to use [36,118,119,120] or consider [121] CytoSorb^®^ in COVID-19. Since initial experience was positive and suggested a therapeutic effect [122], CytoSorb^®^ was temporarily approved in April 2020 by the US Food and Drug Administration (FDA) for emergency use in patients with CRS under certain conditions [123].

Supady and colleagues studied the effect of CytoSorb^®^ therapy in 34 patients with severe COVID-19 pneumonia who received ECMO [124] in a randomized controlled trial. The authors interpreted their data to mean that early initiation of CytoSorb^®^ therapy had a negative effect on survival. Although seemingly well balanced, the study was critizised for incomplete data on treatment details and a survival rate in the control group with ECMO only [73,125] that was much higher than in any other ECMO study. Since no previous study had reported any detrimental effects, hemoadsorption for COVID-19 patients should be used with caution and only in the context of clinical trials.

#### 4.2.6. Post-Pump Syndrome and Perioperative Use in Cardiac Surgery

Cardiopulmonary bypass (CPB) is by principle similar to ECMO, is routinely used in cardiac surgery, and is known to cause a complex inflammatory response with mediator release (C3a, C5a, histamine, IL6, IL8, and TNFα) immediately after blood contact with the foreign surface. Foremost is the activation of the complement and coagulation systems [58,112,126,127]. The SIRS in CPB, termed “post-pump syndrome”, is prognostically relevant [128,129,130] and clinically characterized by increased vascular permeability, decreased peripheral vascular resistance, hypotension and tachycardia, and an increased risk of thrombosis. This suggests that hemoadsorption is reasonable [127,131] and a high ABP may be favourable [72]. Various case series and retrospective studies demonstrated that CytoSorb^®^ application resulted in normalization of cytokine levels, hemodynamic stabilization, reduced vasopressor requirements, less renal replacement therapy, and normalization of serum lactate concentrations [126,132,133,134,135,136].

Two randomized pilot studies on intraoperative CytoSorb^®^ use under CPB failed to demonstrate any advantage for hemoadsorption, which may be due to the small patient numbers, low cytokine levels, and short CytoSorb^®^ application duration [127,130].

In contrast, the multicenter study REFRESH I [58] showed a significant reduction in free hemoglobin, an independent predictor of mortality, on ECMO [137]. Following investigations could not confirm these findings [138].

#### 4.2.7. Intoxications

Due to its binding properties, CytoSorb^®^ is an option in the acute treatment of overdoses or intoxications with various drugs [98] (Table 4). These molecules often contain central hydrophobic structures (e.g., benzene rings) that are adsorbed on the polymer beads even in case of high plasma protein binding [139]. As example, therapeutic levels of rivaroxaban or ticagrelor can be eliminated before emergency surgery [59,140]. Maximization of blood flow rapidly leads to high clearance in time-critical situations. The duration of therapy, blood flow rates, and changing intervals considering the adsorber saturation kinetics should be individually adjusted to achieve optimal clearance [127,141,142,143,144].

**Table 4:** Drugs that can effectively be removed by CytoSorb^®^ or in which a relevant decrease of serum concentrations must be expected (modified from [62]).

	Drug Group	Active Pharmaceutical Substances	References
*positive effect likely*	Antiarrhythmics	Amitriptyline	[145]
		Flecainide	[146]
		Digoxin	[141]
		Digitoxin	[147]
	Antidepressant	Amitryptilin	[145,148]
	Anticonvulsants	Carbamazepine	[141]
		Valproic Acid	
		Phenytoin	
	Beta Blocker	Bisoprolol	[148]
	Calciumchannel blockers	Amlodipine	[148]
		Verapamil	[149]
	Hypnotics and sedatives	Phenobarbital	[141]
	Psychotropic drugs	Quetiapine	[143]
		Venlafaxine	[150]
		3,4-Methylenedioxy-methamphetamine (MDMA, “Ecstasy”)	[151]
	Toxins	Aflatoxine	[32,152]
		Toxic Shock Syndrome toxin-1 (TSST-1)	[32]
		Viper Snake Venom	[153]
*positive or negative effect likely* *(according to the indication)*	Anticoagulants	Dabigatran	[154]
		Edoxaban	[155]
		Rivaroxaban	[59,156]
		Ticagrelor	[59,139,144,156]
	Contrast agents	Iodixanol Iohexol	[157,158]
	Immunosuppressives	Tacrolimus Cyclosporine	[141]
*negative effect likely*	Antibiotics	Amikacin, Vancomycin, Tobramycin, Gentamicin, Ciprofloxacin, Meropenem, Piperacillin, Flucloxacillin, Imipenem, Teicoplanin, Linezolid	[61,63,141,159,160]
	Antimycotics	Fluconazole, Voriconazole	[63]

### 4.3. Side Effects

CytoSorb^®^ is a CE-approved bio- and hemocompatible medical device. Cellular components (e.g., leukocytes/platelets) are minimally extracted. In principle, adhesion of blood cells to the polymer beads is detectable by electron microscopy [138], but the induction of hemolysis or other clinically significant side effects have not been reported [161]. Similarly, there is no evidence for a “CytoSorb^®^-induced” inflammatory response or coagulopathy [138]. CytoSorb^®^ therapy is considered to be safe [72,133]. However, it is to be expected that in patients receiving relevant long-term medication, e.g., anticonvulsants and immunosuppressives, the drug concentrations drop significantly during hemoadsorption [127]. 

Interaction with the CytoSorb^®^ adsorber appears possible from a pharmacokinetic point of view for a variety of the drugs used in intensive care, but requires further investigation. As an example, the adsorption of triiodothyronine [127,162] can be considered. The function as an iodine donor for various immunological pathways is limited with it; e.g., neutrophil granulocyte function is compromised and mortality is increased in patients with low fT3 and fT4 levels in septic shock [163]. Whether hemoadsorption possibly enhances such a pathomechanism has not been investigated until now but cannot be ruled out pathophysiologically.

### 4.4. Dosage of Antibiotics

Antimicrobial chemotherapy is elementary in sepsis therapy and should start within the first hour of diagnosis [9,164]. Anti-infective dosing is very complex and depends on various determinants including hyperdynamic/hypodynamic circulation, volume of distribution, hepatic/renal clearance, albumin concentration, and extracorporeal organ support (ECMO, RRT) [165]. There is an increased risk of mis- or underdosing with hemoadsorption [166,167,168] and dosing needs to be adjusted even under CRRT [169].

CytoSorb^®^ may aggravate this problem by an “anti-antibiotic effect”. Relevant adsorption phenomena (mainly in-vitro data) with high extraction rates [63] have been described for various anti-infectives (Table 4). According to animal data (hemoperfusion over 6h, without adsorber change), the influence of CytoSorb^®^ on the total clearance of the investigated 17 various anti-infectives seems negligible. An additional dose has been recommended for fluconazole and linezolid only [160]. First clinical data for linezolid suggest similar behavior in humans [159]. Due to the initial high volume of distribution and the decrease in clearance within a few hours after CytoSorb^®^ installation, various authors recommend an additional anti-infective dose per adsorber for a variety of antibiotics [127,141,159,170]. Drug monitoring under hemoadsorption therefore seems to be essential [160].

## 5. Perspectives

The interaction of various pro- or anti-inflammatory components of the immune system with different organ systems (cytokine storm) is causative for SIRS, sepsis, and septic shock, the most frequent indications for CytoSorb^®^ use. Modulation of the host defense by “immunomodulation” with a “theoretical” reduction in the plasma concentration of various cytokines and good biocompatibility may potentially help to control shock and prevent multiorgan failure. However, potentially deleterious effects due to the nonspecific interference with complex immunologic processes should be considered. Therefore, hemoadsorption with CytoSorb^®^ as adjunctive therapy in severe cytokine dysregulation states can currently only be recommended in the context of clinical trials. In septic shock, a decrease in mortality under hemoadsorption with CytoSorb^®^ has not yet been clearly demonstrated. Whether a postulated “anti-antibiotic effect” has undesired effects remains speculative. On the other hand, it remains undetermined which patients, if any, could potentially benefit from such a therapy. Possibly, a recently presented dynamic scoring system may be useful to help identifying the right patients [82]. None of these have been scrutinized by adequately powered randomized controlled trials. It is possible that the lack of clear data to date is due to the heterogeneity of the studies published to date. This relates to differences in study type, statistical models, patient populations studied, study design, or primary or secondary outcome. For illustration, some studies on the use of CytoSorb^®^ in septic shock are summarized in Table 5.

Therefore, prospective studies of single indications for clearly defined patient groups, e.g., severe burn patients with cytokine storm and myoglobinemia, under more precisely definable “experimental” conditions would be useful to generate evidence for the respective efficacy or inefficacy of hemoadsorption on the basis of possibly also smaller study cohorts.

Due to its special physicochemical properties, the range of indications for CytoSorb^®^ has been significantly expanded in recent years. Hemoadsorption can be part of complex treatment regimens in liver failure, rhabdomyolysis, or intoxication.

Further high-quality randomized controlled trials of hemoadsorption are urgently needed and should consider factors such as blood flow, dose (amount of blood purified (ABP)), average adsorber use time, and total duration of hemoadsorption (Table 5). Potential side effects or interactions, in addition to a patient population clearly defined by indication and disease severity, should be equally investigated. Based on the findings, relevant contraindications should be formulated. Getting answers to these questions would be a prerequisite for adopting CytoSorb^®^ therapy into routine intensive care medicine.

## Figures and Tables

**Table 1:** External injuries and diseases that can trigger a SIRS potentially followed by Injury-associated Immunosuppression (IAI) or Sepsis Associated Immunosuppression (SAI) modified after [14].

External Injuries	Disease
Polytrauma	Pancreatitis
Craniocerebral trauma	Liver insufficiency
Organ transplantation	Renal insufficiency
Burn	Stroke
Extensive surgery	Myocardial infarction
Cardio-pulmonary resuscitation	Heart failure
Cardiosurgical intervention	Sepsis and Septic Shock

**Table 2:** Cytokine families and their functions with examples of pro- and anti-inflammatory cytokines; modified from [30,31,32].

Family	Functions	Cytokine	Impact on Inflammation	Removal Rate on CytoSorb^®^ (% at 120 min) [30]/[32]
Interferone (IFN)	Regulation of innate immunity; activation of antiviral effects; antiproliferative effects; pyrogenic effect.	IFNγ	Pro	95.7/61
Interleukine (IL)	Growth and differentiation of leukocytes. *Proinflammatory effects*: induction of cyclooxygenase II; expression of various adhesion molecules; NO synthase ↑; pyrogenic effect.	IL1β IL2 IL6 IL7	Pro Pro/Anti Pro Pro	97.2/n.d. 99.3/n.d. 99.6/78 n.d./n.d.
	*Antiinflammatory effects*: inhibition of proinflammatory cytokine (e.g., IL1α, IL1β, TNF) and monocyte/macrophage, Promotion of Th2-lymphocytes	IL1RA IL4 IL10 IL11 IL13	Anti Anti Anti Anti Anti	92.1/n.d. 99.9/n.d. 99.8/n.d. n.d./n.d. 94.2/n.d.
Chemokine	Control of chemotaxis; recruitment of leukocytes; predominantly proinflammatory activity.	IL8 MCP1 MIP-1α	Pro Pro Pro	100/n.d. 100/n.d. 97.3/97.4
Colony-stimulating factors (CSF)	Stimulation of hematopoietic progenitor cell proliferation and -differentiation.	G-CSF	Pro	99.4/n.d.
Transforming growth factors	Regulation of proliferation, differentiation, adhesion of cells.	TGFβ	Anti	n.d./n.d.
Tumor necrosis factor (TNF)	Proinflammatory; activates cytotoxic T-lymphocytes.	TNFα (Cachectin)	Pro	98.4/21.7
Peptide hormone	Early-phase cytokine; uremic toxin; release from myeloid cells; neutrophil migration ↓; phagocytosis performance ↓.	Resistin	Pro	n.d./n.d.
Soluble Cytokine Receptors with Anti-inflammatory Activities	Inhibition of the natural ligands and thus suppression of the typical effect.	sIL-1RII sTNFRp55	Anti	n.d./n.d.

n.d.: no data.

**Table 3:** Indications (para-) clinical criteria for the use of the CytoSorb^®^ adsorber modified from [66,69].

Indications	Clinical Criteria	Paraclinical Criteria
Rhabdomyolysis - Reperfusion syndrome - Trauma - Malignant Hyperthermia	Independent of renal function	Myoglobin > 1000 U/L (observe trend)
Inflammation (SIRS) triggered by: *(1)* External injuries - Sepsis/Septic shock - Hemorrhagic shock ○ Trauma Polytrauma Craniocerebral trauma ○ Ruptured aortic aneurysm - Post-Cardiac Arrest Syndrome - Cardio-pulmonary resuscitation - Extensive surgery ○ Organ transplantation ○ Cardiosurgical intervention - Severe skin and soft tissue damage ○ Burns ○ Necrotizin fasciitis - Post-Cardiotomy Syndrome - Acute Respiratory Distress Syn-drome *(2)* Diseases - Pancreatitis - Liver insufficiency - Renal insufficiency - Stroke - Myocardial infarction - Cardiogenic shock/Heart failure - Tumor Lysis Syndrome - Hemophagocytosis Syndrome	- Norepinephrine > 0.3 µg/kg/min - >1 Vasopressors - additional inotropics	- Metabolic Azidosis (pH < 7.25) - Lactate > 2 mmol/L (observe trend) - (Interleukin 6 > 500 pg/mL)
Liver failure/Hyperbilirubinemia bridging to transplant or to recovery	Icterus	- Total bilirubin > 10 g/dL (observe trend) - MELD > 20
Life-threatening bleeding under Direct Oral Anticoagulants (DOAC)	Medical history (dose, last intake, extent of planned operation) Type of bleeding (major or minor bleeding) Availability of specific antidotes (andexanet alfa)	

**Table 5:** A selection of current studies that have investigated the use of CytoSorb^®^ therapy in sepsis and septic shock. Note the heterogeneity of the studies in terms of study design, patient populations, and outcome parameters. n.d. no data, CS—CytoSorb^®^, RCT—randomized controlled trail, SOFA—Sepsis Organ Failure Assessment Score, APACHE II—Acute Physiology and chronic Health Evaluation Score.

Author	Indication	Study Design	Number of Patients	APACHE II	SOFA (pre)	Procedure	Blood Flow (mL/min)	Adsorber Useful Life (h)	No of Adsorber/Patient	Change Interval (h)	Outcome
Scharf et al. Ann. Intensive Care. 2021 [74]	Septic shock (“cytokine storm”)	Propensity score matching analysis; retrospective	38 with CS. 105 without CS.	n.d.	n.d.	ECMO, RRT, Hemo-perfusion	n.d.	7–12 (Median 9)	1	n.d.	**No difference** between groups
Schultz et al. Journal of Critical Care. 2021 [72]	Septic shock	Retrospective cohort study	70 with CS.	30.2	13.8	CVVHD	100–200	26.75	3.2	24	With high dose **28-d mortality** ↓
Supady et al. Lancet Respir. Med. 2021 [124]	Severe COVID 19-pneumonia with ECMO	Single centre, open-label RCT	17 with CS. 17 without CS.	n.d.	9.0 9.0	ECMO	100–700	24	3	24	**28 d mortality ↑**
Rugg et al. Biomedicines. 2020 [124]	Septic shock	Retrospective study; “genetic” matched analysis	42 with CS. 42 without CS.	n.d.	13.0 12.0	CRRT	n.d.	24 (38 Patients had only 1 CS)	1	24	28 d and in hospital **Mortality** ↓
Kogelmann et al. Journal of the Intensive Care Society. 2020 [104]	septic shock (Pneumonia + ARDS + ECMO)	case series	7	28–56	11–16	CVVHD	100–150	(12)/24	4.14	(12)/24	Observed mortality ↓ vs. predicted mortality
Schitteck et al. Ann. Intensive Care. 2020 [83]	septic shock	Retrospective and prospective cohort study	43 with CS. 33 without CS.	39 35	n.d.	CVVHDF	n.d.	n.d.	n.d.	changed with the CRRT	**No difference** between groups in mortality LOS ICU ↓
Brouwer et al. Crit. Care. 2019 [65]	septic shock	propensity score weighted retrospective	67 with CS. 49 without CS.	n.d.	13.8 12.8	CRRT	250–400	24	n.d.	24	**28 d mortality** ↓
Schädler et al. PlosOne. 2017 [78]	severe sepsis, septic shock + ALI	multicenter RCT	47 with CS. 50 without CS.	24.6 23.8	n.d.	Hemo-perfusion	200–250	6 (for 7 d)	7	24	**no effect**
Kogelmann et al. Crit. Care. 2017 [70]	septic shock	case series	26	27–48	8–20	CVVHD	100–150	(12)/24	2.61	(12)/24	Observed mortality ↓ vs. predicted mortality
Friesecke et al. J. Artif. Organs. 2017 [71]	septic shock	Prospective interventional study	20	n.d.	14.3	CVVH/CVVHD	189 142	~24	n.d.	24	Lactat ↓, Vasopressor ↓ Interleukin 6 ↓

## Data Availability

This article contains only previously published data that have been validated through a peer-review process. No new data were generated or analyzed in this study. Data sharing is not relevant to this article.

## Abbreviations

ABP	Amount of blood purified [L/kg]
ACLF	Acute-on-chronic liver failure
ALF	Acute liver failure
ARF	Acute renal failure
APC	Antigen presenting cell
ARDS	Acute respiratory distress syndrome
COVID-19	Corona-Virus induced Disease 2019
CRS	Cytokine release syndrome
DAMPs	damage-associated molecular patterns
HMGB1	high mobility group box-1 protein
IAI	Injury Associated Immunosuppression
MCP-1	Monocyte chemoattractant protein-1
MDSC	myeloid-derived suppressor cells
NLRP3	Nod-like receptor protein: pyrin domain containing 3
IL	interleukin
RRT	renal replacement therapy
PAMPs	pathogen-associated molecular patterns
PRR’s	Pattern recognition receptors
SAI	Sepsis Associated immunosuppression
SARS-CoV-2	Severe Acute Respiratory Syndrome Corona-Virus 2
SIRS	Systemic Inflammatory Response Syndrome
sIL-1RII	Soluble IL-1 receptor type 2
sTNFRp55	Solube TNF receptor p55
TSST-1	Toxic Shock syndrome Toxin-1
